# The effect and safety of acupuncture on patients with functional constipation: Protocol for a systematic review and meta-analysis of high quality RCTs

**DOI:** 10.1097/MD.0000000000018125

**Published:** 2019-12-10

**Authors:** Mingmin Xu, Wei Zhang, Lu Wang, Xiumei Feng, Ying Li

**Affiliations:** a School of Acupuncture–Moxibustion and Tuina; b Office of Educational Administration; c Graduate School, Chengdu University of Traditional Chinese Medicine, Chengdu 610075, China.

**Keywords:** acupuncture, functional constipation, high quality RCTs, protocol, systematic review

## Abstract

**Background:**

Functional constipation (FC) is a prevalent clinical disease that affects a considerable proportion of the population of all ages. Persistent FC significantly reduces quality of life and influences physical and emotional well-being, as well as consumes many substantial healthcare resources. Acupuncture originates from Traditional Chinese Medicine (TCM), and emerging evidence of several randomized controlled trials (RCTs) published suggest that acupuncture has positive effects for FC. Since 2019, several new results of high quality RCTs about acupuncture treatment for FC have been published. Thus a systematic review will be designed to appraise the effectiveness and safety of acupuncture for improvement of FC in patients based on high quality RCTs.

**Methods:**

We carried out a rigorous literature search in English and Chinese electronic database from inception to present. Two reviewers will identify relevant studies, extract and manage trial information, and then assess the risk of bias in included studies by the Cochrane risk of bias assessment tool. Only high quality RCTs will be included. Data will be synthesized by either fixed-effects or random-effects model regarding to a heterogeneity test. The primary outcome measurement will be the change from baseline in mean complete spontaneous bowel movements and stool form. The secondary outcomes involved disappearance rate of symptoms, proportion of responders, mean transit time, health-related quality of life, and safety of intervention. Meta-analysis will be performed by using Cochrane's RevMan software.

**Results:**

This systematic review will summarize high quality clinical evidence to assess and appraise the effectiveness and safety of acupuncture treatment for FC patient.

**Expected conclusion:**

This systematic review and meta-analysis will provide evidence to determine whether acupuncture treatment is an effective and safe therapy for the prevention and treatment of FC compared with medication treatment.

## 1 Introduction

Functional constipation (FC) is a prevalent clinical disease without any specific physiological changes.^[1]^ Several surveys have reported^[2–5]^ that its prevalence in the general population is approximately 16%, although it can range anywhere from 2% to 27%, depending on the definition used and population studied. According to the Rome IV criteria,^[6]^ FC belongs to chronic and intractable condition, which is characterized clinically by defecatory straining, hard or lumpy stools, a feeling of incomplete evacuation, defecatory obstruction, manual maneuvers to facilitate defecation, and fewer than 3 spontaneous complete bowel movements per week. In addition, FC can lead to other digestive system symptoms such as abdominal pain, gas, nausea, and anorexia, as well as potentially prolonging hospital stay.^[1,6,7]^ Besides digestive system diseases, it may cause disorders in perianal, anorectal lesions, diverticulosis, psychiatric symptoms, and even sudden death due to inducing acute cerebral vascular disease by a rise in blood pressure.^[6–9]^ A study has showed that 89% of constipation patients still reported constipation during follow-up period of more than 1 year.^[2]^

It has been reported that long-term and recurrent constipation causes significant economic burdens both on individuals and societies.^[6,10,11]^ For example, in the USA, FC accounts for more than 2 million visits and about 90 thousand hospitalizations yearly, costing nearly 7 billion USD for diagnostic assessments.^[10,11]^ Hence the management of the FC symptoms is important in improving quality of life for the patients and saving socioeconomic resources.

Because of the unclear etiology and pathogenesis, the therapeutic options are relatively limited. Currently there are currently 3 broad categories of therapies for FC^[8,12]^: non-pharmaceutical, pharmaceutical, and surgical. Lifestyle modification involved dietary changes and a modest increase in aerobic activities is the most widely recommended non-pharmaceutical therapy, but some studies have suggested that the effect is not obviously.^[13,14]^ Pharmaceutical treatments are used to relieve symptoms, such as fiber, osmotic and stimulant laxatives, and selective 5-hydroxytryptamine receptor 4 (5-HT4) agonists, etc.^[15–20]^ But the effectiveness of these therapies is limited and constipation tends to recur when usage of the drugs stops, what is worse, the adverse events result in a long therapeutic course. Surgical treatment has strict indications and is performed only in exceptional cases or reserved for extreme cases of colonic inertia, surgery to treat constipation is not routine.^[21–23]^ Despite different therapies for FC, multiple patients are not completely satisfied with current conventional treatments. And more and more patients with FC commonly seek additional help to look for effective, safe, and non-toxic alternative treatments.^[24,25]^ Nowadays, acupuncture is attractive to both patients and practitioners, which originated from Traditional Chinese Medicine (TCM).^[26]^ Most commonly, acupuncture is accomplished by inserting the thin, stainless steel needles into the acupoints and then stimulating the needles manually or electrically to manipulate the De Qi sensation (a sensation of soreness, heaviness, numbness, or distension).^[27,28]^ Acupuncture has been used for thousands of years to treat a variety of gastrointestinal problems including diarrhea, constipation and gastroenteritis,^[29–31]^ which is effective, safe, and inexpensive to patients. Therefore, acupuncture has become one of the most promising alternative medicine and gains increasing popularity in the world currently. Over the past 10 years, emerging evidence of several randomized controlled trials (RCTs) published in recent years suggest that acupuncture may be an effective treatment for FC, by increasing weekly spontaneous bowel movements, decreasing constipation symptoms, and improving quality of life in FC patients.^[26,32–36]^ However, the results of current systematic reviews did not draw a convincing conclusion to support the clinical value of acupuncture.^[37–40]^ Since 2019, several new results of high quality RCTs about acupuncture treatment for FC have been published.^[26,32–36]^ Thus it is necessary for us to perform a systematic review of the literature to assess the effectiveness and safety of acupuncture for FC based on high quality RCTs.

## 2 Objectives

This proposed systematic review is designed to summarize high quality evidence from comprehensive and up-to-date literature to assess and appraise if acupuncture as an effective and safe therapy in improving functional constipation and preventing relapse and reducing constipation-associated symptoms based on high quality evidence, we may recommend an effective therapy and provided clinical decision scheme for clinicians, and help patients seeking further treatment options.

## 3 Methods and analysis

This protocol is conducted according to the Preferred Reporting Items for Systematic Reviews and Meta-Analysis Protocol (PRISMA-P) statement guidelines and the Cochrane Handbook for Systematic Reviews of Interventions,^[41]^ and the registration number is CRD42019143347 in the PROSPERO.

### 3.1 Inclusion and exclusion criteria

#### 3.1.1 Types of studies

There will be no restrictions on the length of treatment and duration of follow-up. This systematic review will include high quality RCTs in English or Chinese that evaluated the safety of acupuncture and its effectiveness in improving FC, and reducing constipation-associated symptoms, without any date of dissemination or restriction of publication type. To RCTs, it should report adequate randomization methods, eligible diagnosis, eligible outcome measurement, and statistical methods description. Blinding will not be a part of the inclusion criteria because of the particularity of acupuncture manipulation. Exclusion criteria include non-RCTs, quasi-RCTs, retrospective studies, review studies, case reports, uncontrolled trials, and animal mechanism studies.

#### 3.1.2 Types of participants

The adult patients (aged 18 years or older) who have been confirmedly diagnosed with FC (according to Rome II/III/IV diagnostic criteria or guidelines for clinical research) will participant regardless of any information about gender, race, nationality or severity, and duration of disease.

#### 3.1.3 Types of interventions

##### 3.1.3.1 Acupuncture interventions

This review will comprise clinical trials that focus on acupuncture with insertion of needles into selected acupoints up to definite therapeutic depths. The treatment group will be treated with manual acupuncture (MA) or electroacupuncture (EA) based on routine regimens (as the sole treatment or a useful adjunct to other treatments) regardless of the site or type of treatment, needling techniques and stimulation method. Besides, there will be no restrictions in terms of the needle materials, frequency of treatment sessions, and treatment courses. At the same time, auricular acupuncture, fire needling, intradermal needling, 3-edged needling, pyonex, plum blossom needling warm needling, laser acupuncture, dry needling, tap-pricking, point injection, pricking blood, moxibustion, pharmacoacupuncture, transcutaneous electrical nerve stimulation, cupping and acupressure will be excluded. And trials in which acupuncture was compared with different acupuncture points or different forms of acupuncture will be excluded.

##### 3.1.3.2 Comparison interventions

Comparison interventions will be placebo acupuncture, sham acupuncture, no treatment, waiting list membership, usual care, placebo, conventional pharmacological therapies, diet or physical activity therapy, and any other active treatments. In addition, studies that have intervention groups comparing either acupuncture alone with sham intervention alone or acupuncture plus one or more therapies with sham intervention plus the same therapies also will be included.

#### 3.1.4 Types of outcome measures

##### 3.1.4.1 Primary outcomes

The primary outcomes of this review will be the change from baseline in mean complete spontaneous bowel movements and stool form (using the Bristol Stool Form Scale (BSFS)^[1]^).

##### 3.1.4.2 Secondary outcomes

The secondary outcomes involved disappearance rate of symptoms, proportion of responders, mean transit time, quality of life, and safety of treatment assessed by adverse event reporting in studies. The main symptoms include defecation interval time, lumpy or hard stools, soiling and blood-stained stool, sensation of anorectal obstruction/blockage, difficult defecation, and encopresis. Accompanying symptom refers to abdominal pain or flatulence, pain during defecation decreased appetite, dry mouth, halitosis, feverish feeling in palms and soles, and hyperchromic urine. The proportion of responders is defined as the number of responders divided by the total number of participants in each group. Transit time means the time from the first perception of wanting to defaecate to the finish of defaecation, and the mean transit time will also be calculated. The outcome health-related quality of life will be measured by validated tools, such as the Medical Outcomes Study 36-Item Short Form Health Survey (SF-36). And safety that measured by incidence and severity of adverse events associated with using acupuncture in patients with FC.

### 3.2 Search strategy for identification of studies

#### 3.2.1 Electronic searches

Electronic database involving PubMed Database, Cochrane Library, Web of Science database, Embase Database, Medline, Chinese BioMedical Literature Database, China National Knowledge Infrastructure, China Science and Technology Journal database, and Wanfang Data Chinese database from inception to present will be adopted to develop an electronic search strategy. The following search terms will include: “Functional Constipation” OR “Chronic Functional Constipation” OR “Chronic Constipation” OR “Idiopathic Constipation” OR “Slow Transit Constipation” OR “Functional Gastrointestinal Disorder” OR “Functional Defecatory Disorder” OR “Chronic Severe Functional Constipation” OR “Constipation” OR “FC” OR “CC” OR “CSFC” AND “Acupuncture” OR “Acupuncture Therapy” OR “Acupuncture Needle” OR “Manual Acupuncture” OR “Electroacupuncture” OR “Needling” OR “MA” OR “EA”. To ensure the same searching terms in both Chinese and English database, the search words will be translated in the Chinese databases. The search strategy for PubMed is shown in Table 1.

**Table 1:**
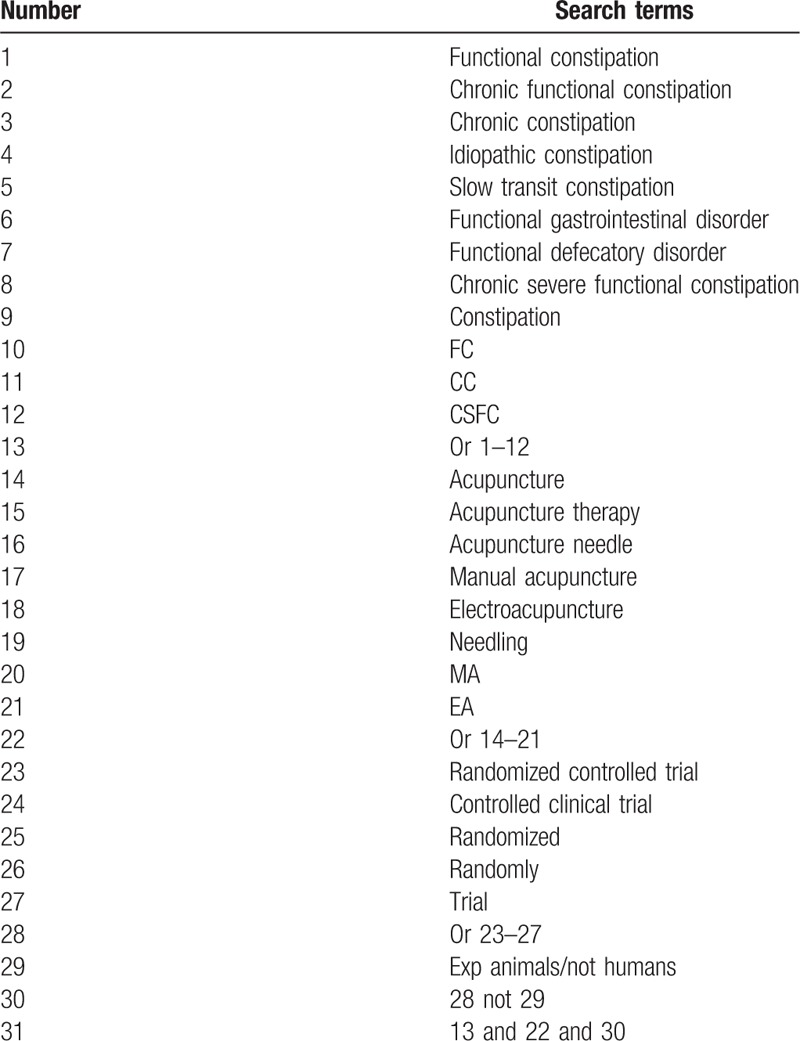
Search strategy for the PubMed database.

#### 3.2.2 Searching other resources

We will also search the clinical trial registries, dissertations, informal publication, and grey literature from inception to the search date. The reference texts of potentially eligible studies, systematic reviews, and relevant conference proceedings related to acupuncture and FC will be retrieved and examined for additional trials. And a list of medical journals will be hand searched in the university library, such as Chinese Medical Journal, etc. Any relevant ongoing or unpublished experimental studies will be gained from the WHO International Clinical Trials Registry Platform (http://www.who.int/trialsearch), metaRegister of Controlled Trials (http://www. controlledtrials.com), United States (US) National Institutes of Health Ongoing Trials Register (http://www.clinicaltrials.gov), Current Controlled Trials (http://www.controlled-trials.com), and the Chinese Clinical Trial Registry (http://www.chictr.org/cn/). Potential grey literature will be elected and searched in OpenGrey.eu. website. We will include relevant conference abstracts, if information is missing we will try to contact the correspondent author to obtain important information and most up-to-date clinical data that are not available through the previously mentioned searching.

### 3.3 Data collection and analysis

#### 3.3.1 Selection of studies

**Figure 1:**
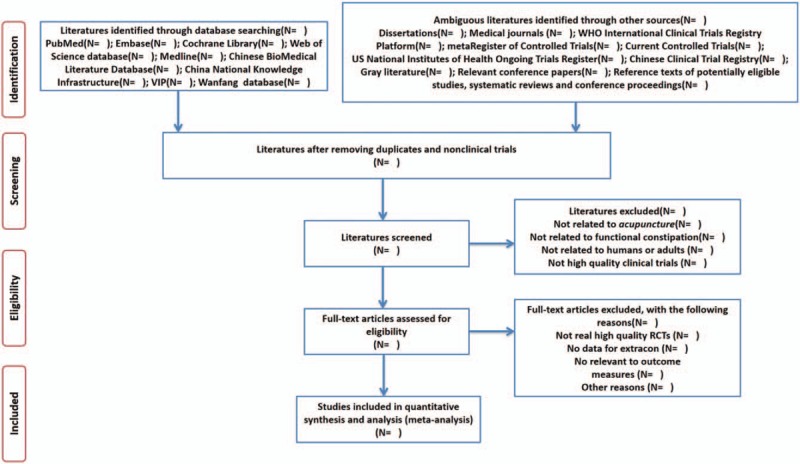
Preferred Reporting Items for Systematic Reviews and Meta-Analyses (PRISMA) flow chart.

All potential relevant clinical studies will be screened according to their titles, abstracts, keywords by 2 reviewers (MMX and LW) at the same time independently after removing duplicates and nonclinical trials. And then the intensive reading of full text could authenticate for further assessment if there are studies that could not be clearly included based on both titles and abstracts. Once any disagreement occurs, a decision will be resolved through discussion among the 2 reviewers (MMX and LW), or argument will be adjudicated by a third reviewer (YL). Details of entire study selection procedure are summarized in flow chart (Fig. 1).

#### 3.3.2 Data extraction and management

Before data extraction, a standard data extraction form (Excel) containing specified outcomes will be created according to the inclusion. Two reviewers (MMX and LW) will then extract data independently such as demographic information, methodological description, population characteristics, acupuncture characteristics, and outcome measures, notes. Any disagreements will be resolved through discussion or consultation between the 2 reviewers if necessary, final determination from a third reviewer (YL) will be sought.

#### 3.3.3 Assessment of risk of bias in included studies

Two reviewers (MMX and LW) will evaluate the risk of bias according to the risk of bias (ROB) tool^[42]^ and completeness of Standards for Reporting Interventions in Controlled Trials of Acupuncture (STRICTA) checklist for reporting intervention details of acupuncture.^[43]^ The risk of bias domains including: selection bias (random sequence generation and allocation concealment), performance and detection bias (blinding of participants and investigators, blinding of outcome assessors and statisticians), attrition bias (incomplete outcome data, differential dropout), reporting bias (selective outcome reporting), and other sources of bias (for example, conflicts of interest, follow-up, the different characteristics and representativeness of participants, the different types of needles used, non-intention-to-treat or per-protocol analysis, etc). In acupuncture, blinding of acupuncturists is impossible because of the nature of acupuncture, but for the studies using sham or placebo acupuncture as a control treatment, the assessment of blinding in both participants and outcome assessors can be conducted. Each trial will be categorized into low bias, unclear (unclear or unknown risk of bias), or high bias. According to the criteria of the Cochrane guidelines, a clinical trial meeting all criteria will be judged as having a low risk of bias, a trial meeting none of the criteria will be judged as having a high risk of bias, and a trial with insufficient information to judge will be classified as unclear risk of bias.

#### 3.3.4 Measures of treatment effect

For dichotomous outcomes, data will be analyzed using the risk ratio (RR) with a 95% confidence intervals (CIs). Other binary data will be changed into the RR form. For continuous data, the weighted mean difference (WMD) or the standard mean difference (SMD) with a 95% CIs will be used. WMD will be used for data measured on the same scales and for which the same assessment instrument is used. SMD will be used if all studies assess the same outcome but measure it in various ways.

#### 3.3.5 Unit of analysis issue

Only the first experimental period date will be considered in randomized cross-over trials. Studies with multiple intervention groups, this review will combine experimental and control intervention groups into a single respectively to avoid a unit of analysis issue.

#### 3.3.6 Dealing with missing data or unclear information

Referring to the Cochrane handbook for systematic reviews of intervention, if there are insufficient details or missing data in relation to the characteristics of the studies, 2 reviewers (MMX and MXF) will attempt to get the information by contacting the authors of the included studies via email or telephone to request missing data or unclear information first. If it is not possible to contact the original authors or obtain sufficient information, we will exclude such studies and only analyze the available data and describe it in the discussion. The potential impact of insufficient data on the findings of the review will be described in Section 4.

#### 3.3.7 Assessment of heterogeneity

If this review includes studies having sufficiently homogenous in terms of design, study population and outcomes recorded on the extraction form, complete data will be regarded as analysis data. Statistical heterogeneity between trial results will be assessed by the Mantel–Haenszel *χ*^2^ texts with a significance level of *P* < .1, and *I*^2^ test will be used for quantifying inconsistency among the included studies. According to the guidelines of the Cochrane Handbook for Systematic Reviews of Interventions, we will set a 50% cut-off point for meaningful heterogeneity among the included studies. In terms of the interpretation of the *I*^2^ statistics, when the *I*^2^ value is less than 50%, the study will not be considered to have statistical heterogeneity. While the *I*^2^ value exceeds 50%, significant statistic heterogeneity exists among the trial and meta-analysis will not be performed. And subgroup analysis will be conducted to determine the reason.

#### 3.3.8 Assessment of reporting bias

If our review has a sufficient number of included trials that are available in the meta-analysis, a funnel plot and statistic test will be generated to analyze the potential reporting bias as well as small study effects.

#### 3.3.9 Data synthesis and analysis

When *I*^2^ < 50% was regarded as no evidence of substantial statistical heterogeneity is detected, and a fixed-effects model will be used for pooled data, when *I*^2^ ≥ 50% was regarded as substantial statistical heterogeneity is observed and a random-effects model will be adopted to synthesize the data and reach a conclusion more cautiously. If the data will not be suitable for combining quantitative synthesis, in this case, a systematic narrative description will be provided with the information that presented in the text to summarize and explain the characteristics and findings of the individual studies.

#### 3.3.10 Subgroup analysis

Subgroup analysis will be conducted to investigate the substantial heterogeneity or inconsistency when sufficient data are available in the included studies. The subgroup analysis will include the factors like the design of the trial, study quality, durations, frequencies, period of follow-up, types or forms of acupuncture intervention (this is the main factor causing heterogeneity), the degree of FC severity, FC measurements, the characteristics of patients, geographical area, time-point of outcomes, and other different control interventions. The incidence rates of different types of adverse events with descriptive techniques will be also tabulated and assessed in this review.

#### 3.3.11 Sensitivity analysis

When there are adequate studies, sensitivity analysis will be adopted for primary outcomes to explore the robustness of conclusions if feasible, and assess the impact of methodological quality, sample size and missing data. Sensitivity analysis will be conducted by removing lower quality studies if heterogeneity remains after subgroup analysis or studies with incomplete results according to the STRICTA checklist. The meta-analysis will be carried out again after trials of lower quality have been excluded. The results of these meta-analyses will then be compared and discussed according to their sample size, strength of evidence and influence on the pooled effect size. However, if all included studies have a high risk of bias, we will not carry out sensitivity analyses.

#### 3.3.12 Grading the quality of evidence

The quality of evidence and confidence for the main outcomes (primary outcomes and adverse events) of including studies in our review will be evaluated and assessed on the basis of the Grading of Recommendations Assessment, Development and Evaluation (GRADE) guidelines.^[44]^ The quality of evidence will be adjudicated into 4 levels: “very low”, “low”, “moderate” or “high” judgment. Any discrepancy will be resolved by consensus or consultation with a third review author (YL).

#### 3.3.13 Ethics and dissemination

This systematic review does not require formal ethical approval given that all the data collected in this study here will not contain individual patient data. The date of any amendment, a description of the change and the rationale in the event of protocol amendments will be documented in the full review. It is our intention to publish results in a peer-reviewed scientific journal and to present our findings at national and international conferences.

## 4 Discussion

FC is a common type of functional gastrointestinal disorders, characterized by persistently difficult, infrequent, or seemingly incomplete defecation excluding Irritable Bowel Syndrome and other organic diseases.^[1]^ The prevalence of FC varies according to diagnostic criteria and region, but the morbidity of FC showed a rising trend year by year with the change of lifestyle.^[2–5]^ The etiology of FC is largely unknown and is probably multifactorial. FC patients with persistent recurrent attacks often experience impaired health-related quality of life and diminished productivity, even increased absences from work, which cause directly and indirectly huge economic loss to patients and the healthcare system.^[6,10,11,45]^ In addition, long-term constipation may induce hemorrhoids and cardiovascular disease, increase the risk of colon cancer, and cause depression and anxiety and other interest exceptions.^[6–9]^ What's more, current treatments for chronic constipation remain unsatisfactory in expected therapeutic effect and produce uncomfortable side effects.^[8,12–23]^ Nowadays, emerging data illustrate that acupuncture is beneficial to improve or even treat FC.^[26,32–36]^ Acupuncture has gained increased popularity in western countries because of its convenience, low cost, safe, and unique therapeutic effects.^[29–31]^ However, there is few of systematic review and meta-analysis about acupuncture for FC published in English literatures. Based on this, our systematic review intends to use evidence-based medicine evidence, search global clinical research on acupuncture treatment of FC, systematically evaluate the efficacy and safety of acupuncture treatment of FC, and provide more scientific evidence for clinical treatment of FC. We sincerely hope the findings will benefit patients with FC and care providers as they will have more treatment options.

Some limitations we must also recognize in this review may be that first, it is difficult to present single or double-blind experiment measures during acupuncture therapy, the participants will know whether they receive acupuncture or medication although they will not know true or sham acupuncture they will receive. Second, diverse style of acupuncture, characteristics of FC patient, and degree of FC severity may cause high statistical heterogeneity. And then we plan to analyze all of the relevant factors in the future to offer more information for clinical practice and scientific research. Third, due to language barriers, this systematic review cannot be searched in more electronic databases, we only selected the included trials in English or Chinese, which may cause some related studies losing.

## Acknowledgments

The authors would like to acknowledge Dr Yu Guo from School of Traditional Chinese Medicine, Jinan University, and Dr Yunzhou Shi from Chengdu University of Traditional Chinese Medicine for providing valuable suggestions to conduct this overview.

## Author contributions

**Conceptualization:** Mingmin Xu, Wei Zhang.

**Data curation:** Mingmin Xu, Wei Zhang, Ying Li.

**Formal analysis:** Mingmin Xu, Lu Wang, Ying Li.

**Funding acquisition:** Ying Li.

**Investigation:** Mingmin Xu, Lu Wang, Xiumei Feng.

**Methodology:** Mingmin Xu, Wei Zhang, Ying Li.

**Project administration:** Mingmin Xu, Wei Zhang, Ying Li.

**Resources:** Mingmin Xu.

**Software:** Mingmin Xu.

**Supervision:** Mingmin Xu, Wei Zhang, Ying Li.

**Validation:** Mingmin Xu, Lu Wang, Xiumei Feng, Ying Li.

**Visualization:** Mingmin Xu, Wei Zhang, Lu Wang, Xiumei Feng, Ying Li.

**Writing – original draft:** Mingmin Xu.

**Writing – review & editing:** Wei Zhang, Ying Li.
